# NEK1 Facilitates Cohesin Removal during Mammalian Spermatogenesis

**DOI:** 10.3390/genes2010260

**Published:** 2011-03-07

**Authors:** Kim Holloway, Elle C. Roberson, Kelly L. Corbett, Nadine K. Kolas, Edward Nieves, Paula E. Cohen

**Affiliations:** 1 Department of Biomedical Sciences, Cornell University, Ithaca, NY 14853, USA; E-Mails: eroberso@uvm.edu (E.C.R.); klc65@cornell.edu (K.L.C.); pc242@cornell.edu (P.E.C.); 2 Department of Biochemistry, Laboratory for Macromolecular Analysis & Proteomics (LMAP), Albert Einstein College of Medicine, 1300 Morris Park Avenue, Bronx, New York, NY 10461, USA; E-Mails: nkkolas@gmail.com (N.K.K.); edward.nieves@einstein.yu.edu (E.N.)

**Keywords:** meiosis, cohesin, kinase, fertility, spermatocyte, mouse, NIMA-like

## Abstract

Meiosis is a highly conserved process, which is stringently regulated in all organisms, from fungi through to humans. Two major events define meiosis in eukaryotes. The first is the pairing, or synapsis, of homologous chromosomes and the second is the exchange of genetic information in a process called meiotic recombination. Synapsis is mediated by the meiosis-specific synaptonemal complex structure in combination with the cohesins that tether sister chromatids together along chromosome arms through prophase I. Previously, we identified FKBP6 as a novel component of the mammalian synaptonemal complex. Further studies demonstrated an interaction between FKBP6 and the NIMA-related kinase-1, NEK1. To further investigate the role of NEK1 in mammalian meiosis, we have examined gametogenesis in the spontaneous mutant, Nek1kat2J. Homozygous mutant animals show decreased testis size, defects in testis morphology, and in cohesin removal at late prophase I of meiosis, causing complete male infertility. Cohesin protein SMC3 remains localized to the meiotic chromosome cores at diplonema in the Nek1 mutant, and also in the related Fkbp6 mutant, while in wild type cells SMC3 is removed from the cores at the end of prophase I and becomes more diffuse throughout the DAPI stained region of the nucleus. These data implicate NEK1 as a possible kinase involved in cohesin redistribution in murine spermatocytes.

## 1. Introduction

Meiosis is a specialized form of cell division that is highly conserved from fungi to humans; beginning with one round of pre-meiotic replication, followed by two rounds of division, to produce haploid gametes for sexual reproduction. The defining stage of meiosis, prophase I [1] encompasses two critical events to ensure faithful completion of meiotic division, namely pairing and synapsis of homologous (maternal and paternal) chromosomes, and the repair of double-strand breaks (DSBs), the latter resulting in the formation of crossover and non-crossovers in a process termed meiotic recombination. Failure to complete synapsis or meiotic recombination within the correct temporal and spatial framework is potentially lethal to the cell and to the organism, since such defects can lead to aberrant chromosome segregation (known as non-disjunction) and consequent production of aneuploid gametes. Thus, it is not surprising that both synapsis and recombination are strictly controlled and highly conserved between species and that mechanisms exist in many species to eradicate cells that show failure in these meiotic processes. That said, however, higher eukaryotes appear to be more susceptible to errors in prophase I than do lower organisms, since aneuploidy rates in humans are 100 to 300 times larger than those seen in yeast [2–5]. Most, if not all, of these aneuploidy events are thought to arise from errors occurring during prophase I.

Synapsis of homologous chromosomes during prophase I is dependent upon the correct assembly of the synaptonemal complex (SC), a tripartite protein structure that starts to form during leptonema of prophase I, when the axial elements localize along the lengths of the homologs, components of which include the SC protein SYCP3 [6] and FKBP6 [7]. During zygonema, the central element begins to form between the homologs, consisting of proteins such as SYCP1, SYCE1 and SYCE2 [8–12], thus tethering them together and ensuring they are paired along their entire lengths at pachynema. Mutations in the genes encoding SC components can result in sterility, or compromised fertility, of knockout mice [6,7,10–12], both as a result of synapsis failure but also because of defects in DSB repair.

In addition to SC formation, homolog synapsis is highly dependent on sister chromatid cohesion (SCC), as mediated by the meiotic cohesins. In mammalian meiosis, there are two levels of cohesion. The first acts along the chromosome arms to maintain cohesion and to ensure homologous pairing until the first division at anaphase I. This cohesion also ensures correct orientation on the meiotic spindle prior to separation. The second level of cohesion at the centromere ensures that sister chromatids remain together until division at meiosis II [13].

SCC is established in pre-meiotic S-phase, and consist of three core subunits; two members of the structural maintenance of chromosomes (SMC) protein family (e.g., SMC1α, SMC1β and SMC3) and proteins from the kleisin family (e.g., REC8 and STAG3) [14–16]. REC8, STAG3, SMC1α and SMC1β all localize to the chromosome cores during prophase I in spermatocytes [14–19]. REC8 localizes to sister chromatids prior to accumulation of cohesins SMC1β or SMC3, or SC components SYCP3 and SYCP2 [17] and persists on the chromosome axes until anaphase I, or at the centromeres until anaphase II, although SC proteins are required for cohesin integrity at diplonema [20]. This suggests that REC8 might ultimately be required for SC axial element formation [21] and subsequent centromere attachment. SMC proteins have been observed to decrease between prophase I and anaphase I, indicating that they are not critical for the completion of meiosis I or for meiosis II (reviewed in [22]). Co-immunoprecipitation studies have indicated the presence of several cohesin complexes in meiosis, and these differing complexes could play cohesion roles on the chromosome arms or at the centromeres.

One component of the SC central element unique to mammals is FK506-binding protein 6 (FKBP6), which is highly expressed in mouse spermatocytes, with expression declining towards the end of prophase I [23,24]. The protein localizes exclusively to chromosome cores as they begin to synapse, and co-immunoprecipitates with SC protein SYCP1 [25]. The intense FKBP6 staining persists into pachynema, and remains until diplonema of late prophase I, when it begins to disappear from the chromosomes, indicating a role in SC assembly/maintenance in mammalian meiosis. Male *Fkbp6*^−/−^ mice are completely sterile, while the females retain full fertility, which is unsurprising given the sexual dimorphism seen in many meiotic mutants (reviewed in [26]). *Fkbp6*^−/−^spermatocytes show severe defects in pairing and synapsis, as detected by accumulation of SYCP3 protein and arrest at pachynema of prophase I [7]. Although its temporal and spatial localization have been defined [7], the exact role FKBP6 in mammalian meiosis remains unclear.

Here, we describe a novel meiotic interaction between FKBP6 protein and a NIMA-related kinase-1, NEK1, a dual specificity serine-threonine and tyrosine kinase. At least 11 orthologs of NIMA-like kinase exist in mammalian species (NEK1-11), although the other family members only possess serine-threonine activity; and have been implicated in many mitotic cellular processes, regulation of the cell cycle and ciliogenesis [27–29]. NEK1 is highly expressed in murine germ cells, predominantly in the testes in spermatogonial cells and in prophase I stage spermatocytes [30,31] and at least two distinct mutant mouse lines for *Nek1*, generated at the Jackson laboratories, display severely impaired fertility [32–34], indicating a probable involvement in meiotic progression. This, coupled with our data implicating NEK1 as a possible FKBP6 interactor, led us to investigate the role of NEK1 in mammalian prophase I progression. Here, we describe in detail the meiotic progression in these mice and the apparent errors in cohesin unloading from the chromosome cores at the end of prophase I.

## 2. Results and Discussion

### 2.1. Nek1^kat2J/kat2J^ Mice Show Decreased Testis Size and Lack Epididymal Spermatozoa

**Figure 1:**
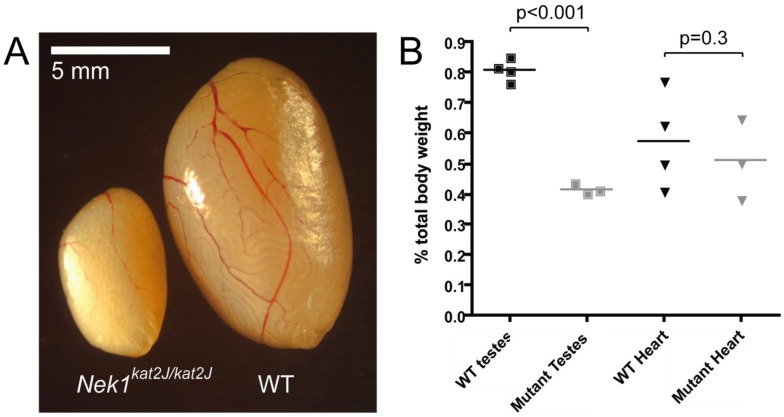
*Nek1^kat2J/kat2J^* mice show decreased testes size. (**A**) Photograph of both *Nek1^kat2J/kat2J^* and wild type littermate testis; (**B**) Wild type (black) and mutant *Nek1^kat2J/kat2J^* (gray) testes and heart sizes, shown as a percentage of total body weights.

Adult testis showed a marked decrease in size in the *Nek1^kat2J/kat2J^* animals, compared with wild type littermates (Figure 1A). Taking into account the smaller body weights of the mutant animals, by measuring testis weight as a percentage of total body weight, the *Nek1^kat2J/kat2J^* males exhibit an average reduction in testis weight of 49% (*p* < 0.0001, unpaired *t*-test, Figure 1B), which is comparable to other meiotic mutants previously characterized [7]. When heart weights were analyzed in the same manner, the hearts of *Nek1^kat2J/kat2J^* mutants were not found to be significantly smaller than those of wild type littermates (*p* = 0.348, Figure 1B). Analysis of sperm numbers from both wild type and *Nek1^kat2J/kat2J^* mutant animals revealed that, while the wild type mice have the expected number of sperm in the caudal epididymides (average of 1.94 × 10^7^ per mouse), the *Nek1^kat2J/kat2J^* mutants have a complete absence of epididymal sperm, indicating severe spermatogenesis defects.

### 2.2. Nek1^kat2J/kat2J^ Mice Show Severe Abnormalities in Testis Morphology

**Figure 2:**
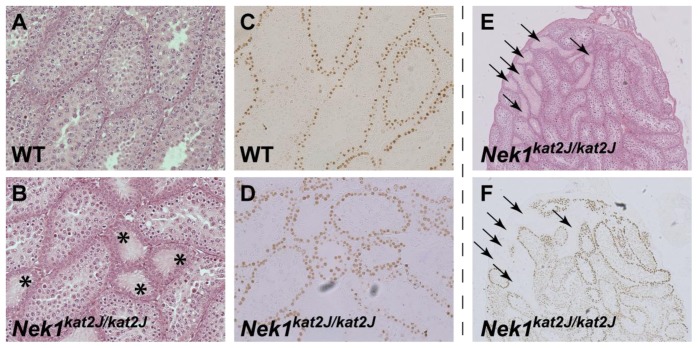
3 week old *Nek1^kat2J/kat2J^* mice show disorganized testes morphology. 3 week old wild type (**A**, **C**) and *Nek1^kat2J/kat2J^* testes (**B**, **D**-**F**) were stained with H&E (**A**, **B**, **E**) or TRA-98 antibody (**C**, **D**, **F**). Empty tubules are shown by the asterisks and empty tubules corresponding to those with no germ cell staining by the arrows.

Histological sections from three-week old wild type and *Nek^kat2J/kat2J^* mutant animals were analyzed by hemotoxylin and eosin (H&E) staining and revealed distinct defects in seminiferous tubule morphology within the testis of the mutant, but not wild type, males. In wild type juvenile animals, the seminiferous epithelium consisted of several layers of spermatogonial cells and the first emerging spermatocyte cells, in addition to the Sertoli cells (Figure 2A). By contrast, however, the seminiferous epithelium of juvenile *Nek1^kat2J/kat2J^* animals showed a heterogeneous appearance, with many tubules being largely devoid of germ cells (Figure 2B, asterisks). To determine if these seminiferous tubules contain any pre-meiotic or meiotic cells, testis sections from juvenile males were stained with an antibody, TRA-98, that recognizes primordial germ cells, type B spermatogonia and spermatocytes [35,36]. The pattern of TRA-98 staining in three-week old testes was not radically different between wild type and *Nek^kat2J/kat2J^* mutant sections in the majority of seminiferous tubules (Figure 2C,D), indicating that spermatogenesis is progressing normally in these seminiferous tubules. However, in those seminiferous tubules with few cells, no TRA-98-positive spermatogonia or spermatocytes were observed, suggesting a failure of pre-meiotic spermatogonial proliferation and/or loss of spermatogonial stem cells.

**Figure 3:**
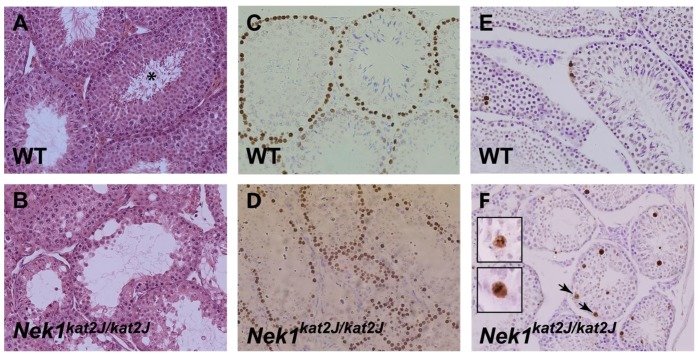
8 week old *Nek1^kat2J/kat2J^* mice show disorganized testes morphology and an increase in apoptotic cells. 8 week old wild type (**A**, **C**, **E**) and *Nek1^kat2J/kat2J^* (**B**, **D**, **F**) testes were stained with H&E (**A**, **B**), TRA-98 (**C**, **D**) or TUNEL (**E**, **F**). The sperm tails in the wild type seminiferous tubules are shown with an asterisk, and the TUNEL positive cells in metaphase by the arrows and in the insets.

By eight-weeks of age, a more dramatic difference in morphology is observed between wild type and *Nek1^kat2J/kat2J^* mutants (Figure 3A,B). The testes of *Nek1*^+/+^ adult males are densely packed with different types of spermatogenetic cells and sperm tails are clearly visible in the lumen of the seminiferous tubules (Figure 3A, asterisk). *Nek1^kat2J/kat2J^* mutant testis sections show much less cellular density within the seminiferous epithelium, with large vacuoles devoid of cells and very few sperm tails in the lumen (Figure 3B). Importantly, however, sperm tails are present, albeit at severely reduced levels.

When stained with anti-TRA-98 antibodies, sections of wild type testis from eight-week old mice show strong labeling of spermatocytes and type B spermatogonia within the seminiferous epithelium, with TRA-98 positive cells being distributed around the periphery of the tubule (Figure 3C). Testis sections from age-matched *Nek1^kat2J/kat2J^* mutant males, however, show extremely disorganized staining of TRA-98, often 3 or 4 cells thick, indicating an increase in early spermatogenic cells within the seminerous tubules of *Nek1^kat2J/kat2J^* males (Figure 3D), possibly as a result of spermatogenic arrest somewhat downstream of the spermatogonial stages.

In order to determine if spermatocytes of the mutant animals are arresting due to apoptosis during prophase I, testis sections from eight-week old *Nek1*^+/+^ and *Nek1^kat2J/kat2J^* males were subjected to TUNEL labeling of apoptotic cells (Figure 3E,F). *Nek1^kat2J/kat2J^* mutant testis contained more TUNEL-positive cells than wild type littermates, approximately a 60% increase compared with wild type littermates, which is highly significant (number of TUNEL-positive cells per tubule 0.98 and 1.56, wild type and mutant, respectively, *n* = 193 and 98, *p* = 0.02). Many of these apoptotic cells appear to be in or around metaphase of meiosis I (Figure 3F, arrows, insets).

### 2.3. Nek1^kat2J/kat2J^ Mice Show Normal Acquisition of Markers of Synapsis and Recombination during Prophase I

**Figure 4:**
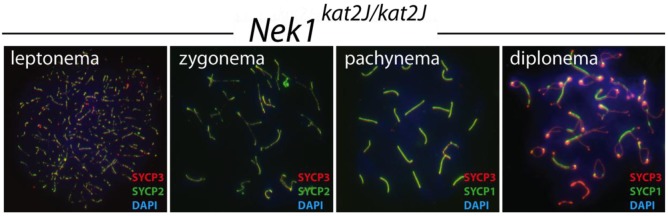
*Nek1^kat2J/kat2J^* spermatocytes undergo normal synapsis. Wild type (not shown) and *Nek1^kat2J/kat2J^* testes were stained with a variety of antibodies against meiotic prophase I proteins. Shown here is staining with SYCP3 (red), either SYCP1 or SYCP2 (green) and DAPI (blue) during the first four stages of prophase I; leptonema, zygonema, pachynema and diplonema.

Prophase I chromosome spreads were stained with antibodies against the SC proteins, SYCP3, SYCP2 and SYCP1 (Figure 4). Chromosome spreads from *Nek1^kat2J/kat2J^* mutant spermatocytes show normal synapsis through early prophase I; they acquire the lateral element proteins SYCP3 and SYCP2 during leptonema, and extend the lateral elements during zygonema. The central element protein SYCP1 accumulates normally during mid prophase, and synapsis culminates in complete co-localization of SYCP3 and SYCP1 along the autosomes at pachynema, comparable with that seen in wild type littermates (data not shown). The SC begins to break down at diplonema, as visualized by SYCP1 protein localization (Figure 4).

**Figure 5:**
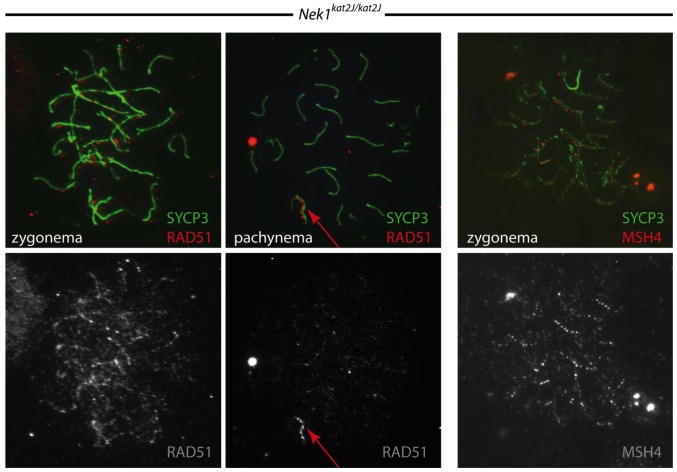
*Nek1^kat2J/kat2J^* spermatocytes undergo normal double-strand break (DSB) initiation and repair. Wild type (not shown) and *Nek1^kat2J/kat2J^* testes were stained with a variety of antibodies against meiotic prophase I proteins. Shown here is staining with SYCP3 (green), and either RAD51 or MHSH4 (red) during the zygotene and pachytene stages of prophase I.

DSB repair pathway components, such as RAD51, MSH4 and MLH1, localize in similar temporal and spatial patterns in both wild type and *Nek1 ^kat2J/kat2J^* mutant spermatocytes, indicating there is no effect of NEK1 loss on DSB repair, *per se*. RAD51 accumulates at leptonema in an expected pattern of localization, while MSH4 localizes to chromosome cores during zygonema (Figure 5). MLH1 localizes on fully synapsed chromosomes during pachynema, at frequencies that are similar between the wild type and mutant mice, again showing no change in crossover frequency (data not shown).

### 2.4. FKBP6 Acts Prior to NEK1 during Mammalian Prophase I

**Figure 6:**
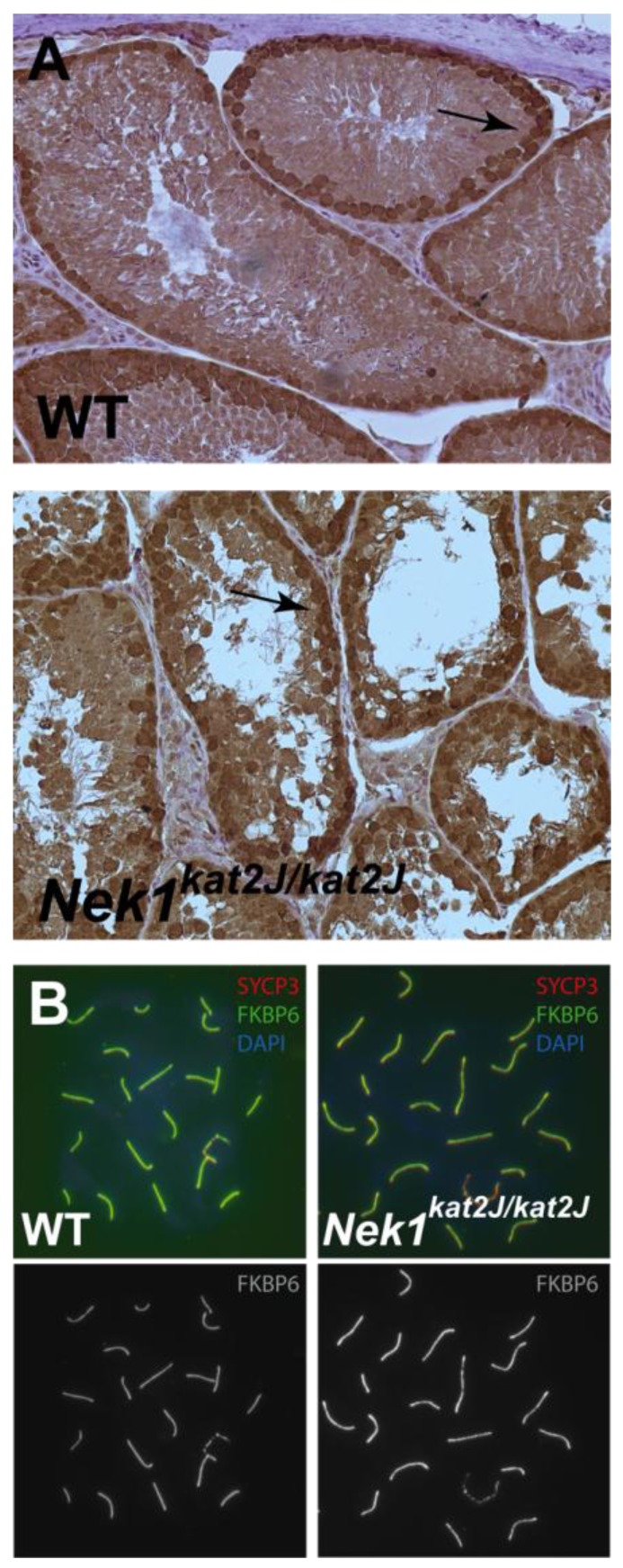
NEK1 protein acts later than FKBP6 in prophase I cells. (**A**) Wild type and *Nek1^kat2J/kat2J^* testes sections were stained with an antibody to FKBP6 (brown stain) and counter stained with Hematoxylin; (**B**) Wild type and *Nek1^kat2J/kat2J^* pachytene spermatocytes stained with anti-SYCP3 (red), anti-FKBP6 (green) and DAPI (blue).

**Figure 7:**
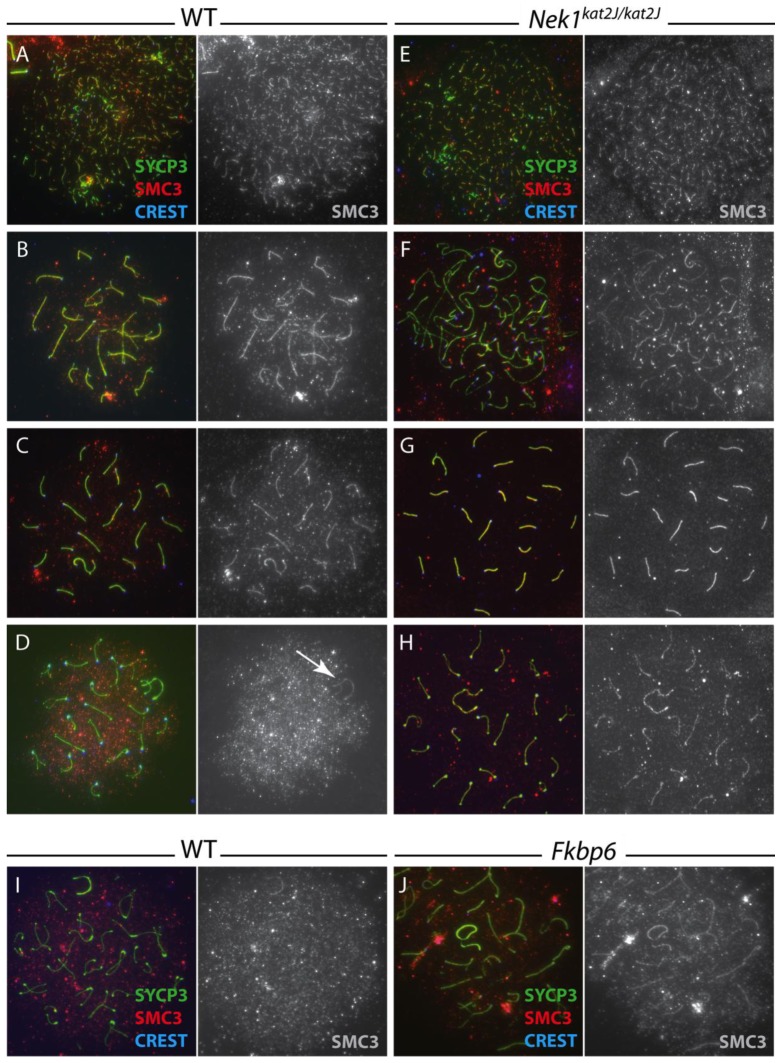
Cohesin protein SMC3 is not removed in the proper temporal manner in *Nek1^kat2J/kat2J^* spermatocytes. Wild type (A–D) and *Nek1^kat2J/kat2J^* (E–H) spermatocytes were stained with anti-SYCP3 (green), anti-SMC3 (red) and CREST autoimmune serum (blue). Cells in leptonema (A, E), zygonema (B, F), pachynema (C, G) and diplonema (D, H) were assessed for SMC3 localization (gray panels show SMC3 staining alone). The persistent XY chromosome staining of SMC3 in wild type cells is shown by the arrow. Wild type (I) and *Fkbp6^−/−^* (J) spermatocytes were also stained with anti-SYCP3 (green), anti-SMC3 (red) and CREST autoimmune serum (blue), however only the diplotene stage is shown here.

NEK1 interacts with the SC component, FKBP6 (data not shown). To explore this interaction further, eight-week old wild type and *Nek1^kat2J/kat2J^* mutant testis were stained with an antibody against FKBP6. Similar localization of FKBP6 was noted in both wildtype and mutant testes (Figure 6A), indicating that the loss of NEK1 protein has no effect on the chromosomal localization of FKBP6 protein in primary spermatocytes. This would suggest that NEK1 acts downstream of FKBP6 or, at the very least, that localization of FKBP6 is not dependent on functional NEK1. Further evidence for this comes from chromosome spread staining of prophase I spermatocytes from wild type and *Nek1^kat2J/kat2J^* mutant mice, using antibodies against SYCP3 and FKBP6; there was a very similar staining pattern observed in both wild type and *Nek1^kat2J/kat2J^* mutant cells (Figure 6B and [7]). On spread preparations from both wild type and *Nek1^kat2J/kat2J^* males, FKBP6 localizes to the chromosome cores in a similar manner to the central element component SYCP1, only along regions of full synapsis in late zygonema and pachynema. The same staining pattern of FKBP6 in both wild type and mutant spermatocytes again suggests that NEK1 is acting downstream of FKBP6, with the absence of NEK1 protein having no notable effect on FKBP6 localization. Taken together, these results indicate that FKBP6 is acting prior to NEK1 during prophase I.

### 2.5. NEK1 may Play a Role in Cohesin Removal in Prophase I Spermatocytes

The meiotic cohesin complex consists of REC8, STAG3, SMC1α and SMC1β, all of which localize to the chromosome cores during prophase I in spermatocytes [14,16–19]. REC8 localizes to sister chromatids prior to accumulation of cohesins SMC1β or SMC3, or SC components SYCP3 and SYCP2 [17] and persists on the chromosome axes until anaphase I, or at the centromeres until anaphase II, although SC proteins are required for cohesin integrity at diplonema [20].

To assess cohesin localization in both wild type and *Nek1^kat2J/kat2J^* mice, antibodies against various cohesin components were applied to prophase I chromosome spread preparations (Figure 7A–H). The localization of cohesin protein SMC3 in *Nek1^kat2J/kat2J^* cells (Figure 7E–G) is similar to that seen in wild type (Figure 7A–C) from leptonema through to pachynema, co-localizing with the SC component SYCP3 on the chromosome cores. During diplonema, however, the SMC3 staining in the wild-type spermatocytes becomes diffuse across the cell, no longer localizing to the cores, except on the X and Y bivalent (Figure 7D, arrow), where the SMC3 remains localized to the chromosome cores. By contrast, chromosome spreads from *Nek1^kat2J/kat2J^* testes show persistent SMC3 distribution along the cores, co-localizing with SYCP3 (Figure 7H). This staining pattern indicates a major role for NEK1 in removal of meiotic cohesin SMC3 from homologous chromosome cores at the end of meiotic prophase I, in preparation for the first meiotic division.

To determine whether the same staining patterns of SMC3 were also seen in spermatocytes from *Fkbp6*^−/−^ spermatocytes, chromosome spread preparations were obtained from testes of *Fkbp6*^+/+^ and *Fkbp6*^−/−^ adults and stained with anti-SMC3 antibodies (Figure 7I,J). Similar to the *Nek1^kat2J/kat2J^* mutant spermatocytes, the spermatocytes from *Fkbp6*^−/−^ males display normal SMC3 localization from leptonema through to pachynema (data not shown), however the *Fkbp6*^−/−^ diplotene cells also retain SMC3 on the chromosome cores, unlike that seen in *Fkbp6*^+/+^ littermate control animals. These data support the idea that the FKBP6-NEK1 pathway is involved in cohesin removal.

**Figure 8:**
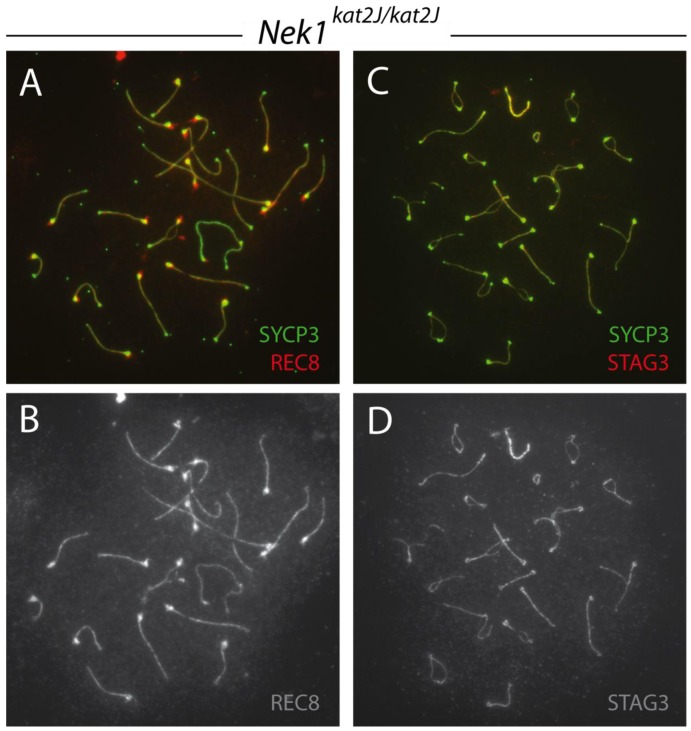
REC8 and STAG3 cohesin localization in *Nek1^kat2J/kat2J^* spermatocytes. *Nek1^kat2J/kat2J^* prophase I spermatocytes were stained with anti-SYCP3 (green) and either anti-REC8 (A, B), or anti-STAG3 (C, D) (red or gray).

When stained with antibodies to other cohesin components, meiotic spreads from *Nek1^kat2J/kat2J^* mice showed no difference to those from wild type mice. Cohesins REC8 (Figure 8A,B) and STAG3 (Figure 8C,D) showed similar staining patterns in both the wild type and mutant cells.

**Figure 9:**
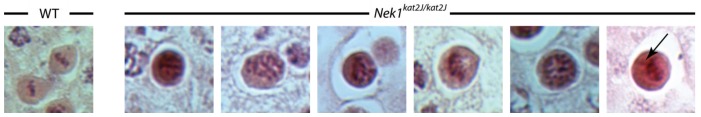
Chromosomes fail to align on the metaphase spindle correctly in *Nek1^kat2J/kat2J^* spermatocytes. *Nek1^kat2J/kat2J^* and wild type testis sections were stained with H&E, and cells undergoing metaphase I imaged. Wild type cells generally show proper alignment of chromosomes on the spindle (left), whereas the majority of cells in *Nek1^kat2J/kat2J^* testis look abnormally aligned or have lagging chromosomes (arrow).

Spermatocytes from testis sections that were undergoing metaphase I were examined by H&E staining from both wild type and *Nek1^kat2J/kat2J^* animals. An increase in misaligned chromosomes was seen in the *Nek1^kat2J/kat2J^* testes, compared with the proper alignment on the spindle seen in the majority of wild type cells (Figure 9). In addition, *Nek1^kat2J/kat2J^* testes showed more cells that had lagging chromosomes than their wild type littermates (Figure 9, arrow).

Female *Nek1^kat2J/kat2J^* mice are reported to be fertile [32], however previous reports and our own experiments failed to yield litters from *Nek1^kat2J/kat2J^* females. This is more likely due to the fact that very few *Nek1^kat2J^* mutants survive beyond two months of age, and those that do display severe growth retardation and other developmental defects, along with, often fatal, polycystic kidney disease [33,34]. We performed a simple analysis of ovaries from 21-day old *Nek1^kat2J/kat2J^* females, and compared them to wild type littermates. Although the ovaries were comparatively much smaller in the mutants, there is evidence of a number of primary oocytes present, indicating that these mice as far as meiosis I with no difficulties. This is not surprising, given the sexual dimorphism previously described for other meiotic mutations [7,37], and given the fact that different cohesion complexes are present in male and female gametes [38]. Alternatively, the persistence of oocytes could be due to a lower stringency checkpoint in female than in male meiosis [39] or to differences in SCC control in males versus females [40]. Either way, the question arises as to how cohesin disassembly is affected in the absence of NEK1 in these persistent oocytes.

Here, we describe a full analysis of fertility and prophase I progression in *Nek1^kat2J/kat2J^* male mice. The fact that these mice have small testis size and no spermatozoa within the caudal epididymis indicates severe defects in spermatogenesis, which is consistent with our TUNEL staining data suggesting an increased amount of apoptosis at or around metaphase of meiosis I. We see severe defects in histology of testes from *Nek1^kat2J/kat2J^* males, displaying a decrease in cellularity and very few sperm tails in the lumen of adult testis. Surprisingly perhaps, considering the interaction with FKBP6, we see normal accumulation of synaptonemal complex and DSB repair proteins in *Nek1^kat2J/kat2J^* spermatocytes. However, towards the end of prophase I, during diplonema, the *Nek1^kat2J/kat2J^* spermatocytes, as well as *Fkbp6* mutant spermatocytes, show defective removal of cohesin protein SMC3. Immunofluorescent and immunohistological analyses indicate that FKBP6 is acting upstream of NEK1 in mouse spermatogenesis, which is consistent with our previous reports of cells not progressing as far as pachynema in some cases in *Fkbp6* spermatocytes [7]. By contrast, *Nek1^kat2J/kat2J^* females appear to undergo normal prophase I progression, as adult ovary sections show normal follicle development, again consistent with the phenotype seen in *Fkbp6* females (data not shown).

These data represent the first in depth analysis of the infertility phenotype of *Nek1^kat2J/kat2J^males*, and reveal that juvenile male *Nek1^kat2J/kat2J^* animals show a delay in entry into meiosis in some tubules. This is evidenced by the lack of cellularity within some seminiferous tubules of juvenile homozygous mutant males. This paucity of cells within certain tubules may be the result of pre-meiotic errors in replication or the transition from S-phase, or may be a result of a smaller pool of spermatogonia within the testes of *Nek1^kat2J/kat2J^* males, but it is hard to distinguish between these possibilities given the observed phenotypes. However, by adulthood, all tubules contain cells that have entered meiosis, and these cells appear to begin prophase I normally, indicating that the pre-meiotic error does not have a bearing on whether the cells enter prophase I.

*Nek1^kat2J/kat2J^* males show severe defects in cohesin SMC3 removal during diplonema, late in prophase I. It has been previously reported in yeast that cohesins are removed prior to anaphase I, in a “step-wise” manner, which is dependent upon phosphorylation of Rec8 [41] and a similar mechanism is proposed in mammals [42]. We propose that NEK1 may be a player in this pathway, given that a cohesin component (SMC3) is not removed from the chromosome cores in *Nek1^kat2J/kat2J^* males. Whether NEK1 is working directly upon REC8, or whether it acts further downstream, is as yet unknown, however given that NEK1 is expressed in all tissues of the mouse [43], we believe it is unlikely to be acting directly upon the meiosis-specific REC8 protein, unless it has different target proteins in different cell types in mammals. Previous studies of the dynamics of cohesin localization in meiotic cells have shown that, although SMC3 protein disperses from the chromosome cores in late prophase I, the timing of this removal may differ between male and female meiocytes. In female oocytes, SMC3 dissociation from the chromosome cores occurs at late diplonema or diakinesis, coincident with SC disassembly [40,44]. In wild type mammalian spermatocytes, on the other hand, SMC3 appears to persist on the axial elements of the SC throughout prophase I, and disassociates from the chromosome arms only towards the end of diplonema/beginning of diakinesis, remaining bound to the centromeres until the second meiotic division [44,45]. In our hands, SMC3 dissociates from the chromosome cores of wild type spermatocytes slightly earlier, at diplonema, but remains localized more diffusely throughout the nucleus. This same staining pattern is also seen in wild type spermatocytes stained during our *Fkbp6* experiment, giving us confidence that this pattern is different from that seen in wild type controls and that FKBP6 and NEK1 are both responsible for correct unloading of cohesin SMC3, perhaps even acting in the same pathway.

Contradictory staining patterns have been described for another meiotic cohesin component in mammalian spermatocytes, SMC1α, where one report described localization on homologous chromosome cores through to the end of diplonema [45], while another suggested that SMC 1α dissociates from meiotic chromosome cores before the end of diplonema [14,46]. There can be many reasons for these slight discrepancies; the use of different antibodies or batches of the same antibody, or differences in chromosome spread preparation protocols may both affect epitope binding. Further evidence that cohesin proteins may be disassembled slightly earlier during diplonema comes from studies of RAD21/SSC1 localization on mouse spermatocytes during prophase I [47]. RAD21/SCC1 is the regulatory subunit of mitotic cohesin, as it links together all the other subunits of the cohesin complex, and its dissociation from the chromosome cores results in the removal of other cohesin subunits also. RAD21/SCC1 is present on all chromosome cores until diplonema, when its localization is severely decreased [47], indicating that cohesin components are being removed from the arms of homologs at this time during prophase I. Interestingly, RAD21/SCC1 is differentially localized at the sex chromosomes and autosomes in prophase I cells. Staining of the X and Y is reported as faint during early pachynema, but increases to a stronger intensity during late pachynema/early diplonema [47], which is interesting given the persistence of SMC3 staining only on the sex chromosomes of wild type spermatocytes in our experiments.

Further evidence for SC components having an effect on cohesin disassembly in meiotic cells comes from the *Sycp3* mouse mutant, which shows premature disassembly of the cohesin complex at the diplotene stage of prophase I [20]. These data provide strong evidence that SYCP3 is required for maintaining SCC during meiosis I. Conversely, the transverse filament protein of the SC, SYCP1, protein is not absolutely required for the correct association of cohesin from the chromosomes in meiosis I [10], although we do not know whether it is required for SCC disassembly as the vast majority of spermatocytes lacking SYCP1 arrest earlier in prophase I.

Other factors have also been shown to be important in the timely removal of SCC components at the end of prophase I, including the multi-protein anaphase-promoting complex/cyclosome (APC/C) [13]. The APC/C is ultimately responsible for controlling the degradation of cohesins in both meiosis and mitosis at the metaphase- anaphase transition, by the direct targeting of cell cycle components, such as securin, PLK1, aurora kinase B and cyclin B. Although this pathway has not been proven to involve the kinase NEK1 thus far, these data do indicate that kinases play a vital role in the degradation of cohesin, allowing homologs or sisters to separate.

## 3. Experimental Section

### 3.1. Animals and Genotyping

We obtained a line of NEK1-deficient mice harboring a spontaneous frameshift mutation in the *Nek1* gene, termed *Nek1^kat2J^*, from the Jackson laboratory (Bar Harbor, Maine). All animal care procedures were approved by institutional animal care and use committee (IACUC). Mice were genotyped by PCR using primers 26508F 5' GCCTGACTCAAAAGCAGGAC 3' and 27111R 5' CATCGGAACTCTTGTGGTGAC 3' followed by subsequent sequencing with primer 27111R. Genotypes were ascertained by the presence or absence of the G base pair insertion in exon 7. For the purposes of these studies, homozygous mutant animals (*Nek1^kat2J/kat2J^*) were compared with wild type (*Nek1*^+/+^) littermates throughout.

### 3.2. Sperm Counts

Epidiymides were removed from either mutant or WT adult mice, placed in human tubular fluid (HTF) culture medium containing BSA (Specialty media), ripped open using micro forceps and the contents squeezed into the medium. The spermatozoa were cultured for 20 min at 32 °C, then a 20 μL aliquot was removed and fixed in 480 μL 10% formalin. The fixed cells were gently mixed then intact spermatozoa counted using a hemocytometer.

### 3.3. Histology

Adult mice were subjected to either perfusion fixation with Bouins fixative or the testes were removed and fixed in 10% buffered formalin for 12 h at 4 °C. Paraffin-embedded tissue was sectioned at 4 μm and processed for Haematoxylin and Eosin staining or immunohistochemical analyses using standard methods.

### 3.4. Chromosome Spread Analysis

Testes were removed from adult mutant and WT mice for the meiotic time course analysis, as well as adult mice for the focus counts, and processed as previously described [48]. Briefly, testes were removed and decapsulated into hypotonic sucrose extraction buffer (HEB, containing 1.7% sucrose) and left on ice for 60 min. Tubules were macerated on glass depression slides in a bubble of 0.03% sucrose and added to slides coated in 1% paraformaldehyde. The slides were dried slowly in a humidified chamber for 3 h and washed in PBS containing Photoflo (Kodak, EMS).

### 3.5. Immunofluorescence and Immunohistochemistry

Slides were processed as described previously [49] using antibodies generated in this lab [48], generously donated by colleagues and available commercially. Immunohistochemistry was performed on formalin-fixed sections using rat hybridoma supernatant against germ cell nuclear antigen-1 (TRA-98) [35] for staining of early spermatocytes, rabbit anti-FKBP6 antibody or TUNEL staining (Chemicon) to detect cells undergoing apoptosis.

### 3.6. Mass Spectrometry

Whole testis protein was extracted from mice by sonication in RIPA buffer (50 mM Tris, 150 mM NaCl, 0.1% SDS, 0.5% Naa.Deoxycholate, 1% NP40, supplemented with 100 μg/mL PMSF and 1× Complete protease inhibitor (Roche). Briefly, 1 mg of whole testis protein was pre-cleared with protein A/G beads (Santa Cruz Biotechnology) and then incubated with an antibody against FKBP6 at 4 °C overnight. Fresh protein A/G beads were added for the final 2 h of the incubation. The protein lysate was then centrifuged and the supernatant removed. The remaining beads were washed in fresh cold RIPA buffer three times and the final bead slurry was resuspended in 40 μL 2× SDS protein loading dye. The protein samples were run on a polyacrylamide gel. The lane was cut into equal sized pieces and sent for mass spectrometry analysis.

Electrospray LC-MS/MS was performed on a QqTOF mass spectrometer, QStar Pulsar i (AB/Sciex, Foster City, CA, USA). An Ultimate Plus nano-HPLC system with a Famos autosampler (Dionex Corporation, Sunnyvale, CA, USA), was coupled to the microionspray source. Peptides were loaded on a C18 μ -PrecolumnTM Cartridge (5 μ m, 100Å, 300 μ m i.d. × 5mm) from the autosampler with a 25 μL sample loop at a flow rate of 15 μL/min. After injection of sample, 20 μL, and washing for 20 min, the precolumn was switched in line with the analytical column, a C18 PepMap100, 3 μm, 100 Å, 75 μm i.d. × 150 mm (Dionex Corporation, Sunnyvale, CA). Mobile phase B (80% acetonitrile/water + 0.1 % formic acid) was increased from 2 to 55% over 70 min, held for 5 min, increased to 95% over 20 min and held at 95% B for 5 min. The flow rate used was 5 μL/min and mobile phase A consisted of 5% acetonitrile/water + 0.1% formic acid. The three most intense ions having a charge state between +2 to +5, determined from an initial survey scan from 300–1800 m/z, were selected for MS/MS. The MS/MS files were converted to text files and searched with Mascot (Matrix Science; http://www.matrixscience.com) against the MSDB mouse protein database. The following parameters were used: trypsin, 2 missed cleavages; variable modifications of carbamidomethylation (Cys), deamidation (Asn and Gln) and oxidation (Met); monoisotopic masses; peptide mass tolerance of 3.0 Da; product ion mass tolerance of 0.6 Da. Proteins were considered identified having at least one bold red (BR) peptide (the most logical assignment of a peptide to a proteins and prevents duplicate homologous proteins to be reported). Only 1 significant protein (*p* < 0.05) was detected, trypsin, the enzyme used for digestion. Thus, indicating the low amount of starting material. Other low probability identified proteins, each with only 1 BR peptide are: protein kinase NEK1, S25284, -MESLKAQTNARAAVLK; axonemal dynein heavy chain 5, Q8VHE6, AITPENIHREVSFNTLDTADGGLLNSVR; and monoclonal antibody 12/131/93 immunoglobulin heavy chain variable region (fragment), AAC82376, IDPANGNTKSDPK. A blast search against all species of the NEK1 and dynein matching peptides resulted in *E* values of 1e-05 and 2e-17, respectively. Despite these low confidence mass spectrometric results we decided to investigate these proteins further.

### 3.7. Statistical Analyses

Testis weights, spermatozoa numbers, TUNEL analysis and immunofluorescent focus counts were all analyzed for statistical significance by using an unpaired *t*-test.

## 4. Conclusions

While NEK1 appears to be largely essential for progression through the first meiotic division, it is important to note that some spermatids were observed in the testes of *Nek1^kat2J/kat2J^* males, suggesting that these cells were able to proceed to meiosis II. At least a fraction of these cells progress to at least the elongating spermatid stage, because we observed a very small proportion of sperm tails in the tubular lumen, but we presume that the majority of these cells cannot proceed beyond meiosis II. If NEK1 acts, not only during SMC3 cohesin removal in meiosis I, but in its removal from the centromeres at meiosis II also, this could account for the continued loss of spermatids as a result of incorrect segregation of chromatids at meiosis II.

These data, together with previous data concerning control of cohesins during yeast meiosis, offer an exciting new insight into a possible novel member of the pathway responsible for control of cohesin removal and subsequent proper segregation in mouse spermatogenesis. We provide further evidence here that SC associated proteins, such as FKBP6, play an important role in cohesin-core organization during mammalian meiosis, and that this may be ultimately controlled by the kinase activity of NEK1. Further studies will be performed to assess the role of NEK1 kinase activity on potential serine-threonine and/or tyrosine containing substrates, and the impact of these activities on cohesin dissolution during meiosis.
